# Genetic analysis of wild walnuts in Xinjiang based on whole-genome resequencing

**DOI:** 10.3389/fpls.2025.1645319

**Published:** 2025-12-19

**Authors:** Yuyu Liu, Ping Zhang, Zhuli Wang, Jilong Fu, Jing Han, Yihang Gao, Zhibo Feng

**Affiliations:** College of Forestry and Landscape Architecture, Xinjiang Agricultural University, Urumqi, China

**Keywords:** whole-genome resequencing, Xinjiang wild walnut, genetic relationship, genetic differentiation, *Juglans regia* L.

## Abstract

**Introduction:**

As a treasured wild plant resource in the Tian shan Mountains, the genetics and evolutionary relationships of Xinjiang wild walnuts (*Juglans regia* L.) are of great interest for both walnut conservation and crop improvement.

**Methods:**

In this study, a total of 200 walnut accessions, including a core germplasm collection of wild walnuts from Xinjiang and local walnut landraces and cultivars, were selected for whole-genome resequencing, with the final dataset supplemented with 24 other publicly available genomic datasets for other walnut taxa.

**Results:**

Across all samples, there was evidence of four ancestral genetic populations, with three of these represented in the samples from Xinjiang. The Xinjiang wild walnuts form an independent evolutionary clade with low genetic diversity, which was further differentiated into six subgroups, and showed significant genetic differentiation from the cultivated accession. The walnut cultivars and landraces showed mixed ancestry, being assigned to two ancestral populations not represented in the wild walnuts. The Gongliu Wild Walnut Valley served as one of the refugia during the Last Glacial Maximum (LGM) for Tertiary relict species. The unique topography of the Ili River Valley in Xinjiang, along with the relatively isolated geographical location of the Walnut Valley, may have collectively facilitated the formation of a relatively isolated “genetic island” pattern in the Xinjiang wild walnuts. Selective sweep analysis identified 20 genes under selection, including *CYP450* genes closely associated with disease resistance and *NF-YB3* genes involved in cold stress and other adaptive responses.

**Discussion:**

A new framework is needed to reconceptualize the genetic relationships of Xinjiang wild walnuts with other germplasms, clarifying their continuous role throughout the evolutionary continuum from glacial refugium to domestication and modern breeding.

## 1 Introduction

Walnuts (*Juglans regia* L.) are perennial deciduous trees in the Juglandaceae family, valued as an important economic crop. Notably, their seed kernels contain a high oil content of 60%–70%, earning them the designation “oil-rich tree (Zhao et al., 2018; Shi et al., 2022). Beyond their economic value, walnuts are rich in ω-3 fatty acids, antioxidants, and high-quality proteins, which have potential beneficial roles in health maintenance and disease prevention (Li, 2007). Xinjiang is an important walnut producing area in China, with a unique ecology and rich walnut genetic resources. As a widely cultivated nut crop, the evolutionary history and genetic diversity of cultivated walnuts have been studied in depth; however, few studies to date have systematically examined the population genetic structure of wild walnuts in Xinjiang, or how the wild walnuts are related to other walnut germplasm.

Xinjiang wild walnut (*Juglans regia* L.) is a valuable Tertiary-relict temperate broadleaf forest species (Wei et al., 2023) that is found primarily in the Wild Walnut Valley Nature Reserve in Gongliu County, Xinjiang, China (Zeng, 2005). During the last glacial period, wild walnuts survived in small refuges located across the Tianshan Mountains to Ferghana Ridge and Southern Kazakhstan, a region spanning 30°N to 45°N (Aradhya et al., 2017). Northern Xinjiang therefore likely contained walnut refugia, and extant wild walnut populations in Gongliu are also located within this geographical region. As such, the Xinjiang walnut populations likely contain unique germplasm and are of particular interest to researchers (Ye et al., 2024). To date, several phenotypic analyses (Wang et al., 1997, 1998; Zhang et al., 2011, 2012, 2013, 2014) and molecular marker-based genetic analyses (Li et al., 2010; Rui, 2010; Wang et al., 2015) have assessed the diversity of Xinjiang germplasm. The study of population genetic structure is essential for analyzing adaptive evolution and genetic relationships in different walnut populations (Gunn et al., 2010). However, very few published studies examining the evolutionary history of walnut have included Xinjiang wild walnut (Ding et al., 2022). In those that have, so few samples of wild Xinjiang materials were included that the studies are not representative of the wild population as a whole, conclusions based on this small sample must be considered tentative (Luo et al., 2022), and estimates of its genetic diversity may be inaccurate. Some studies have suggested that wild walnut in Xinjiang is the direct ancestor of cultivated walnut (Lin et al., 1984; Wang et al., 2015). In addition, Xinjiang wild walnut has been variously described as *Juglans regia* L (Han et al., 2024), *Juglans cathayensis* Dode (Zhang, 2016), and *Juglans fallax* Dode (Zeng, 2005). The taxonomic status of wild walnut in Xinjiang is therefore poorly resolved, and additional genetic studies of the Xinjiang germplasm, evaluating its relationship to other walnut taxa, are urgently needed. Until such studies are performed, the unique and valuable wild walnut resources in Xinjiang will remain poorly utilized and protected.

The study of genetic diversity and genetic structure of wild walnut is important for the conservation and utilization of walnut species (Li et al., 2024). In this study, 200 walnut accessions collected from the Ili, Aksu, Kashgar and Hotan regions of Xinjiang were subjected to whole-genome resequencing. The samples included 79 wild walnut accessions, 103 walnut landraces, and 18 cultivated walnut varieties; additional genomic data for 24 other walnut taxa were obtained for a larger comparative analysis. This study provides essential genomic data for Xinjiang walnut, allowing the elucidation of relationships among wild walnut populations within Xinjiang itself, as well as between wild walnuts and other walnut germplasm.

## 2 Materials and methods

### 2.1 Plant materials and DNA extraction

**Figure 1:**
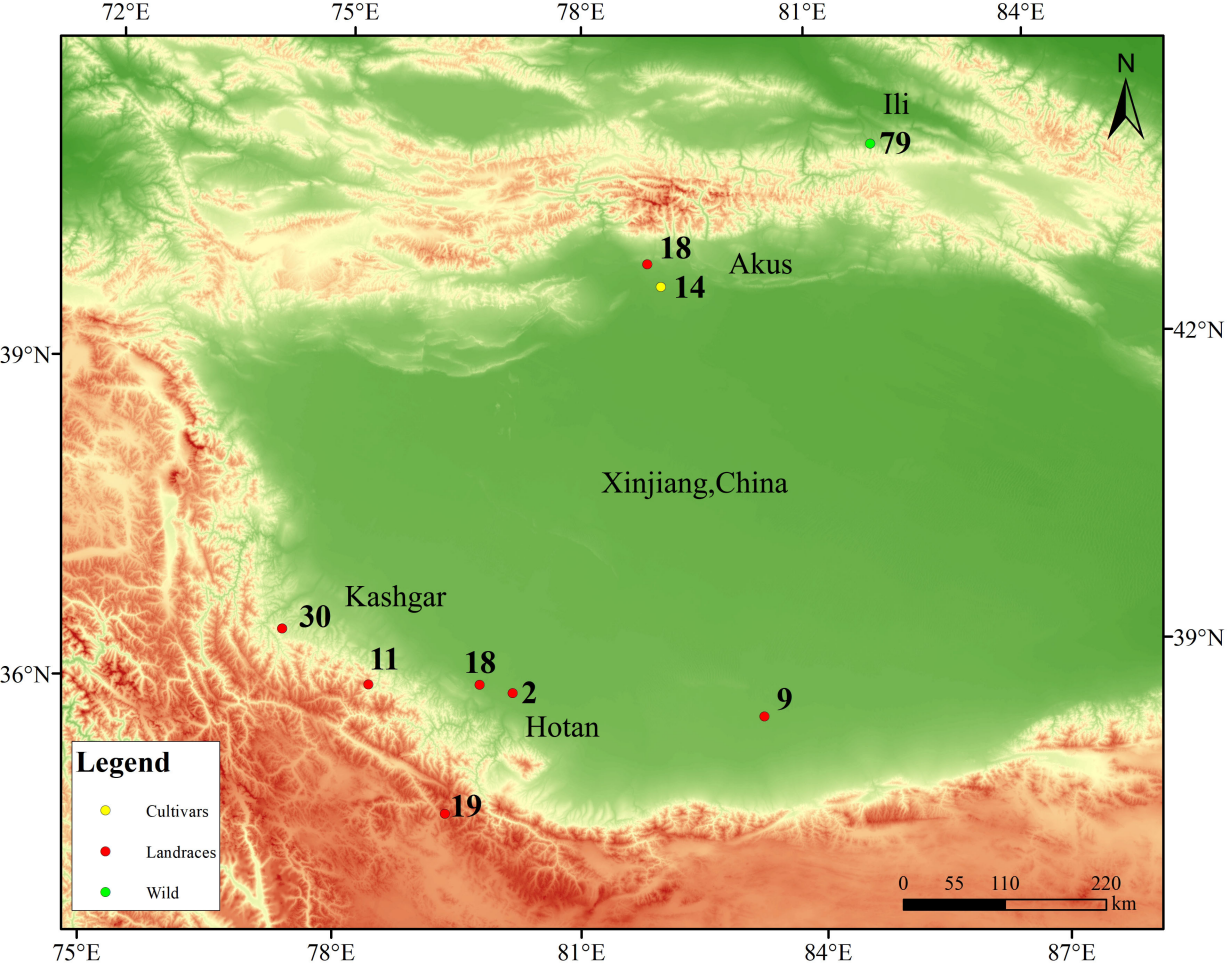
Sample site distribution map. Green circles represent wild walnuts, red circles represent landraces, and orange circles represent cultivars.

Plant tissue samples were collected from natural walnut forests in the Wild Walnut Valley Nature Reserve located in Gongliu County, Xinjiang, China, with the coordinates of 43°19’N-43°23’N, 82°15’E-82°17’E, and the altitude of 1250–1700 m (**Figure 1**). The total area of the reserve is about 10.19 km², and there are more than 5,500 mature trees of wild walnut. Building on previous work by our project team that used SSR and SRAP markers to analyze genetic diversity and establish a core germplasm collection for wild walnuts in Xinjiang, this study systematically assembled a representative set of 79 accessions, including accessions with cold and disease resistance (Yuan, 2012; Yuan et al., 2012; Zhang, 2015; Zhang et al., 2018; Wen et al., 2022, 2023). In addition, samples were collected from 103 individual trees belonging to ancient walnut landraces in Xinjiang, including 59 accessions from Hotan (HTMF, HTMY, HTPS, and HTX), 30 accessions from Kashgar (KSYC), and 14 accessions from Aksu (AK18-AK31), as well as from 18 cultivated walnut varieties local to Xinjiang (AK1-AK17 and AK32). Healthy, fresh young leaves were collected from each accession (n = 200 in total) and immediately placed in liquid nitrogen after collection.

The final dataset was supplemented by 24 publicly available walnut genomic datasets which were obtained to more comprehensively analyze phylogenetic relationships within walnut. The publicly available data comprised eight genomes published by Zhang et al. in 2019 (Zhang et al., 2019a) and Cao et al. in 2023 (Cao et al., 2023): *Juglans cathayensis* (n = 3), *Juglans mandshurica* (n = 2), *Juglans sigillata* (n = 2), and *Juglans hopeiensis* (n = 1). These were supplemented by 15 genomes published by Ji et al. in 2021 (Ji et al., 2021): JR2 (n = 9), JR3 (n = 4), and *Juglans sigillata* (n = 2) (see **Supplementary Table S1** in the **Supplementary Materials** for an overview of all walnut samples included in the study). The geographic distribution of all sample locations was illustrated using ArcGIS, and elevation data for each location were downloaded from a geospatial data cloud website.

DNA was extracted from the leaf tissue samples using a CTAB-based method (Doyle and Doyle, 1987). The quality of the isolated genomic DNA was verified through both the use of agarose gels and a NanoDrop spectrophotometer, as follows: (1) DNA degradation and contamination were assessed on 1% agarose gels; and (2) DNA concentrations were measured with a ND-2000 (NanoDrop Technologies). Only high-quality DNA samples (*OD*_260/280_ = 1.8-2.0, *OD*_260/230_≥2.0) were used to construct sequencing libraries.

### 2.2 DNA sequencing and data quality controls

A total of 0.5 μg of DNA per sample was used as input material for DNA library preparation. Sequencing libraries were generated using a Truseq Nano DNA HT Sample Prep Kit (Illumina USA), with individual barcodes added to each sample. Briefly, genomic DNA samples were fragmented via sonication to a size of 350 bp. The resulting DNA fragments were end-polished, A-tailed, and ligated with full-length adapters for Illumina NovaSeq X Plus sequencing, followed by further PCR amplification. After purification of the PCR products [AMPure XP system (Petrova and Angel, 2024)], sequencing libraries were analyzed to assess their size distribution using an Agilent 2100 Bioanalyzer and quantified using real-time PCR (3nM). The final paired-end libraries were sequenced on an Illumina NovaSeq X Plus system by the Shanghai Majorbio Bio-Pharm Technology Co., Ltd.

The raw sequencing data contained both low-quality base calls and missing values, which would greatly interfere with subsequent data analyses. To obtain clean reads, the following filters were applied. Raw reads of low quality (mean phred score< 20), including reads containing adapter fragments and/or unrecognizable nucleotides (N base > 10), were trimmed or discarded using fastp (Chen et al., 2018).

### 2.3 SNP calling and filtering

To generate BAM files, quality-filtered reads were mapped to the walnut reference genome using *BWA-MEM* (Jung and Han, 2022) with default mapping parameters, the version of the reference genome used was *Juglans regia* Version 1.0 (Zhang et al., 2020). As a modified GATK Best Practice (McKenna et al., 2010), the alignment (BAM) files were sorted using samtools (Li et al., 2009) and PCR duplicates marked with MarkDuplicates. After base quality recalibration, variant calling (for both SNPs and InDels) was performed across all samples using the Haplotyper and Gvcftyper algorithms in the Sentieon Genomics Tools package (Freed et al., 2017). Variants were filtered using standard hard filtering parameters following the GATK Best Practices pipeline (McKenna et al., 2010). Variant functional annotation was performed for the quality-filtered SNPs using *SnpEff* (Cingolani et al., 2012) in combination with gene predictions from the walnut reference genome. Both SNPs and InDels were categorized based on their chromosomal positions (i.e., intergenic regions [including 1-kb upstream and downstream], exons, introns, splice sites, and untranslated regions) and on their effects (i.e., missense, splicing, start codon gain/loss, and stop codon gain/loss mutations). Prior to further analysis, several SNP filtering steps were performed to reduce false positives for genotype calling: (i) SNPs with more than two alleles were removed; (ii) SNPs with a mean depth value over all samples of less than four were removed; (iii) SNPs with a minor allele frequency< 0.05 were removed; (iv) only SNPs that could be genotyped in at least 70% of the samples were retained; and (v) linkage disequilibrium (LD) pruning was performed in *PLINK* prior to population structure analyses (Purcell et al., 2007). Using the final SNP set, further analyses were carried out to assess walnut genetic diversity, kinship parameters, linkage disequilibrium, and population structure, among other investigations.

### 2.4 Phylogenetic tree and population structure analysis

Maximum likelihood (ML) and neighbor-joining (NJ) phylogenetic trees were constructed using *IQ-TREE 2* (model GTR+I+G4; 1,000 bootstraps) (Minh et al., 2020) and *FastTree* (model -gtr -gamma; 1,000 bootstraps) (Price et al., 2010), respectively, based on the LD-pruned SNP set. An unsupervised ML clustering algorithm, as implemented in *ADMIXTURE* (Alexander et al., 2009) was used to estimate shared ancestry in the study walnut populations. Initial clustering was performed for K = 1 to K = 20 ancestral groups with default settings. To maximize the accuracy of the initial clustering, LD-pruned SNPs were used for all structure analyses.

### 2.5 Linkage disequilibrium and genetic diversity analyses, genetic differentiation analysis

To evaluate LD decay across the walnut genome, the squared correlation coefficients (*r*^2^) between pairs of SNPs were calculated using *PopLDdecay* (Zhang et al., 2019b). The average *r*^2^ value was calculated for each pair of SNPs within a 500 kb region and then averaged across the genome. To comprehensively assess the genetic diversity and population structure of the study walnut populations, Stacks (Catchen et al., 2011) was used to calculate population-based divergence indices. The genetic diversity of each locus was estimated based on parameters such as the expected heterozygosity (He), inbreeding coefficient (Fis), nucleotide diversity (π), observed heterozygosity (Ho), polymorphic information content (PIC), and Shannon diversity index (H). Together, these parameters were used to assess the structure of genetic diversity within and among the walnut populations included in the study, providing insight into the evolutionary history of the Xinjiang wild walnuts. To understand the degree of differentiation between populations, the differentiation index *F*_ST_ was calculated using a formula. The calculation formula for the *F*_ST_ of a single locus is as follows:


$$\hat{\theta }=\frac{s^{2}-\frac{1}{2\bar{n}-1}\left[\bar{p}(1-\bar{p})-\frac{r-1}{r}s^{2}\right]}{\left[1-\frac{2\bar{n}C^{2}}{(2\bar{n}-1)r}\right]\bar{p}(1-\bar{p})+\left[1+\frac{2\bar{n}(r-1)C^{2}}{(2\bar{n}-1)r}\right]\frac{s^{2}}{r}}$$

Here, $\bar{n}$ represents the average sample size per population, C is the coefficient of variation in sample sizes across populations, $\bar{p}$ is the mean allele frequency across all populations, $s^{2}$ denotes the variance in allele frequencies among populations, and $\hat{\theta }$ denotes Weir & Cockerham’s unbiased estimator of *F*_ST_. The value of *F*_ST_ ranges between [0, 1], with values closer to 1 indicating greater genetic differentiation among populations.

### 2.6 Principal component analysis and genetic relatedness analysis, selective sweep analysis

To visualize the genetic relationships among samples, a principal component analysis (PCA) was performed based on the LD-pruned SNP set using *PLINK* (Purcell et al., 2007). *PLINK* was also used to estimate degrees of kinship (Manichaikul et al., 2010) between all study individuals based on pairwise SNP comparisons. Using a sliding window approach, genetic diversity (*π*) and genetic differentiation indices (*F*_ST_ and *d_XY_*) were calculated using PIXY (Korunes and Samuk, 2021) for windows of 10 kb; measures of selection (i.e., Tajima′s *D*) were calculated using VCFtools (Danecek et al., 2011). Additionally, *ChiPlot* (https://www.chiplot.online/) (Xie et al., 2023) was used to create a heat map illustrating genetic differentiation (*F*_ST_) among the various walnut populations. Only the window with average value above the threshold of the 95% confidence interval was considered as the candidate selected region.

## 3 Results

### 3.1 Sequencing data overview and variant identification

To understand the genetic relationships among wild and cultivated walnuts in Xinjiang, China, at total of 79 wild walnut accessions, 103 walnut landraces, and 18 cultivated walnut varieties were selected for whole-genome resequencing (350 bp, paired-end reads). After filtering the raw sequencing data, a total of 1,676.33 Gb of high-quality, clean data was obtained. In terms of sequencing quality, the Q30 value averaged 96.22%, ranging from 85.25% to 100% for individual samples. The average GC content was 37.36%, ranging from 36.38% to 41.35% for individual samples. On a per sample basis, the amount of raw data ranged from a minimum of 5.68 Gb (AK15) to a maximum of 26.59 Gb (JC3), with an average efficiency of 99.05%. Following alignment to the reference genome, the average sequencing depth was 14.21 across all samples; on average, 94.26% of the genome was covered at 1X and 86.17% was covered at 4X. More than 95% of the samples had a match success rate of 98%. This rate reflects how similar the sample sequencing data are to the reference genome; the coverage and sequencing depth may also directly reflect homology with the reference sequence. Here, similarity to the reference genome was sufficient to allow the accurate identification of variants and meaningful assessments of the Xinjiang walnut population.

**Figure 2:**
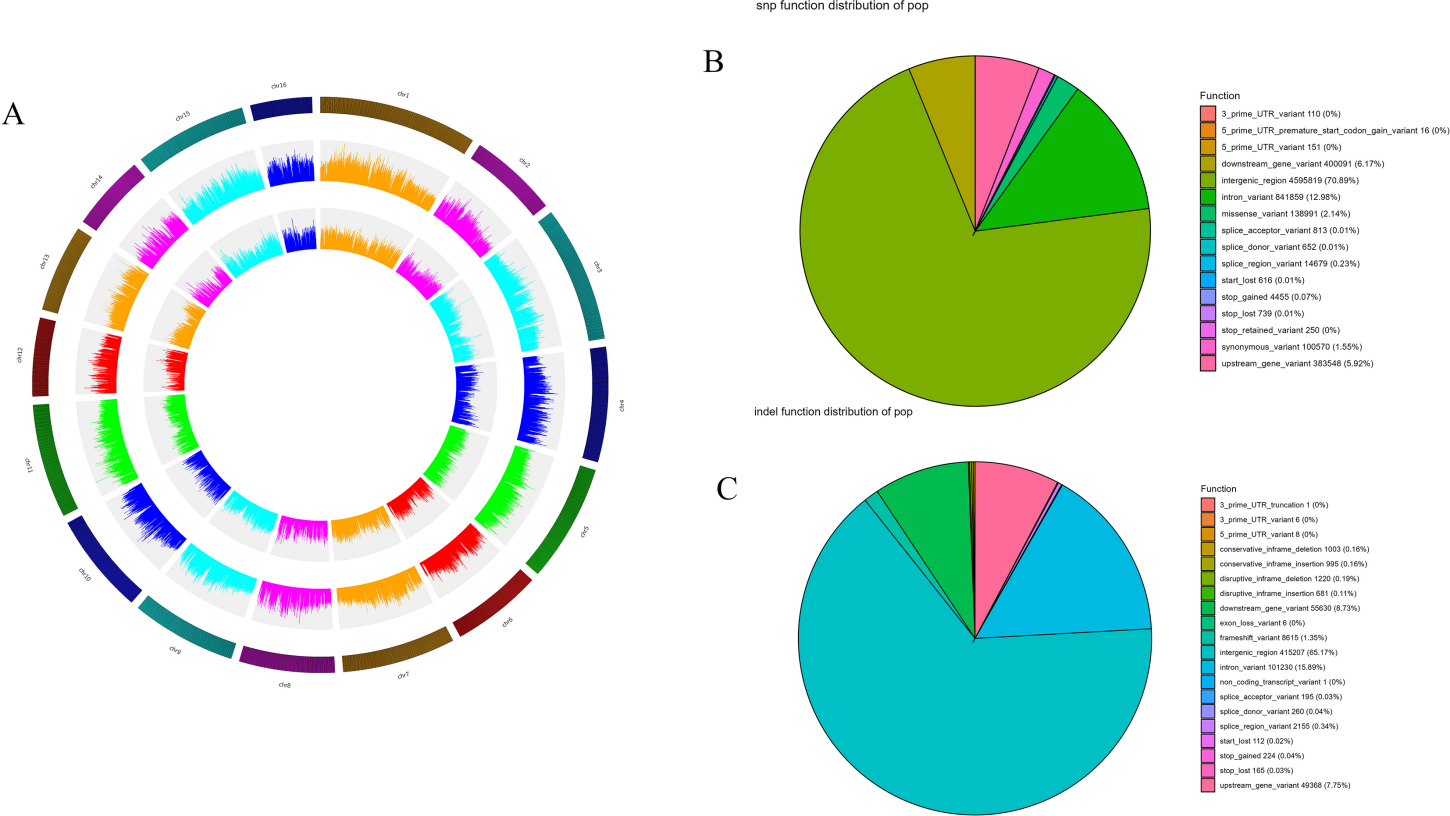
**(A)***Circos* plot illustrating the distribution of genes (outer ring), SNPs (middle ring), and InDels (inner ring) along the chromosomes of the walnut genome for the 224 walnut accessions; the color depth (heatmap) and bar height (histogram) both illustrate the density of variants. **(B)** Pie chart of the functional effects of SNP loci in the walnut genome. **(C)** Pie chart of the functional effects of InDel loci in the walnut genome. All analyses are based on the full set of 224 accessions.

A total of 30,477,372 SNPs were identified across the walnut genome. After filtering, 5,679,611 high-quality SNPs were retained for analysis, as well as 5,905,456 insertion-deletion polymorphisms (InDels). Among the InDels, 3,135,605 were insertions and 2,769,851 were deletions. All variants were mapped using *Circos*, as illustrated in **Figure 2**, which visually presents the distributions of SNPs, InDels, and other variants across the chromosomes of the walnut genome. Variant-rich regions may contain important gene functions and/or be subject to natural selection.

### 3.2 Genetic diversity and linkage disequilibrium in genomes, genetic differentiation analysis

Nucleotide diversity (π) was calculated to understand patterns of genetic diversity in the walnut germplasm from Xinjiang. The nucleotide diversity of all walnut samples measured 0.286 at the whole-genome level. The lowest nucleotide diversity was found in the wild walnut population from Xinjiang (0.252), which was significantly lower than that of walnut landraces (0.308) and cultivated walnut (0.305) (**Table 1**); the highest nucleotide diversity occurred in the Hotan landraces. Similarly, the Shannon diversity index was highest in cultivated walnut (0.457), followed by the landraces (0.447) and wild walnut population (0.371) (the genetic diversity indices of the populations are presented in **Supplementary Table 2**). Differences in SNP and InDel frequencies revealed genetic differentiation between wild and cultivated walnut samples from Xinjiang. The population segregation (*F*_ST_) index measures among-population differentiation by assessing allele frequency differences between populations. Pairwise *F*_ST_ values between wild walnuts and landraces were higher, demonstrating greater genetic differentiation than that between cultivated walnuts and landraces, based on both SNP and InDel datasets (the *F*_ST_ values between clusters are provided in **Supplementary Table 3**). Looking more broadly across all walnut populations, the Xinjiang wild walnuts were the most genetically distinct, showing substantial differentiation from both landraces (average *F*_ST_ = 0.076) and cultivated walnuts (*F*_ST_ = 0.072). Conversely, walnut cultivars and landraces were the least differentiated (*F*_ST_ = 0.014), underscoring their close genetic affinity.

**Figure 3:**
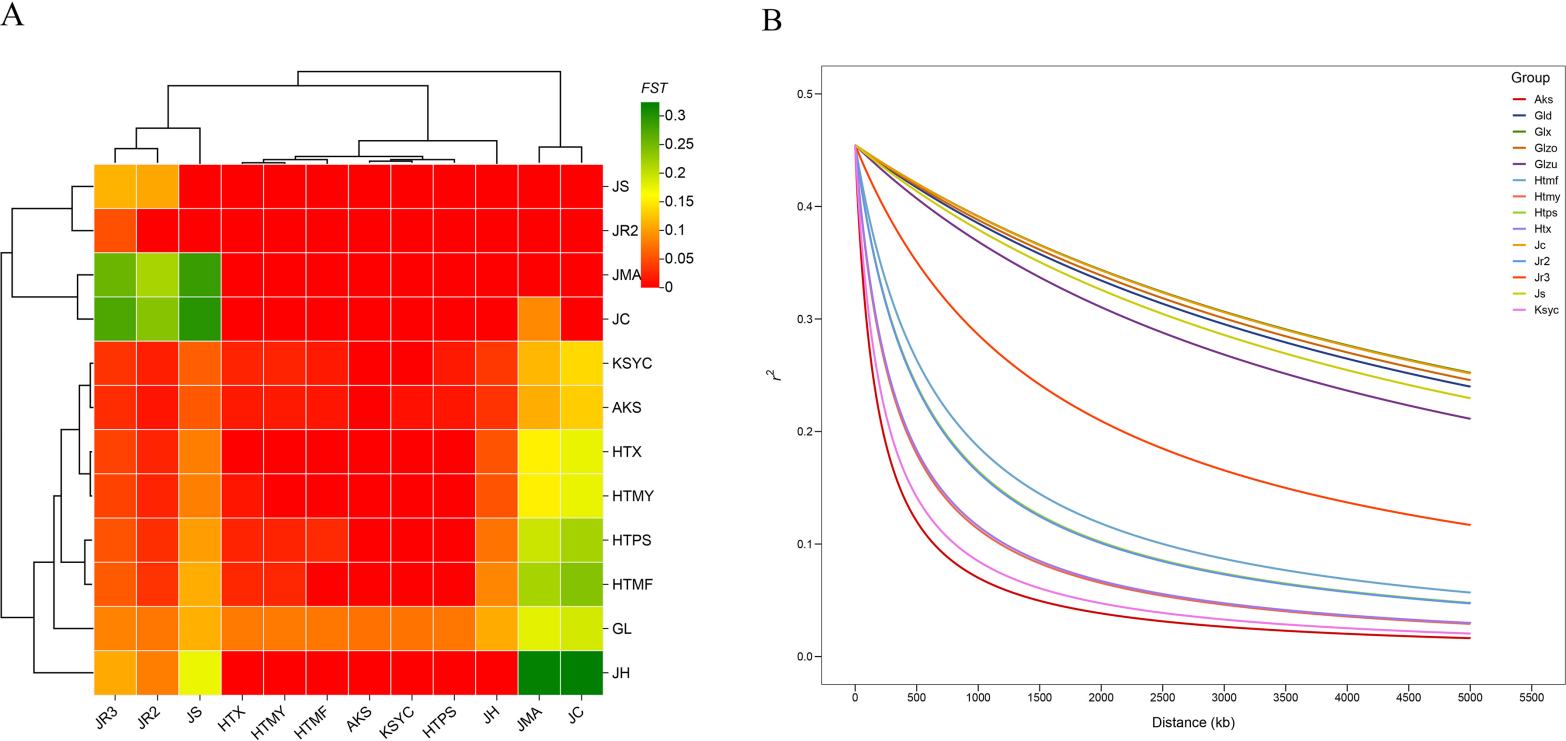
**(A)** Heatmap illustrating pairwise *F*_ST_ values between walnut populations, where *F*_ST_ is a measure of among-population genetic differentiation. Population names are provided on the horizontal and vertical axes, and the phylogenetic relationships among populations are also illustrated on the sides. **(B)** Linkage disequilibrium decay plot with trends for individual walnut populations plotted separately. The distance between pairs of SNPs is plotted on the *x*-axis, while *r*^2^ values are shown on the *y*-axis. Population codes: GL (wild walnuts, incl. Glzu, Glzo, Gld, Glx); HTX, HTMY, HTPS, HTMF, KSYC (ancient seedlings); AKS (cultivated AK1-AK17, AK32; ancient seedlings AK18-AK31); JC, JH, JMA, JR2, JR3, JS (24 publicly available accessions).

Linkage disequilibrium (LD) is a critical concept in population genetics and evolutionary biology, and the extent of genetic differentiation among populations, as well as the evolutionary history, genetic diversity, and genome structure of individual populations, may all influence LD levels. In this study, a LD analysis was performed for all walnut populations. The wild population exhibited a slower LD decay rate than other populations (**Figure 3B**), which corresponded to a considerably larger LD half-decay distance in the wild population (approximately 1 Mb) compared to landraces (173.582 kb) or cultivated walnuts (91.67 kb). The slow decay of LD in Xinjiang wild walnuts might be explained by the geographical proximity of samples. The LD decay rates of cultivated varieties and landraces were similar. In AKS, HTMY, HTPS, HTX, and KSYC, LD decayed rapidly over short physical distances, suggesting that these populations had high recombination rates and genetic diversity. In contrast, in the wild group, LD remained high over long physical distances, suggesting a low recombination rate and lower genetic diversity, perhaps due to bottleneck events and/or geographic isolation.

### 3.3 Phylogenetic relationships and population structure among wild and cultivated walnuts in Xinjiang

**Figure 4:**
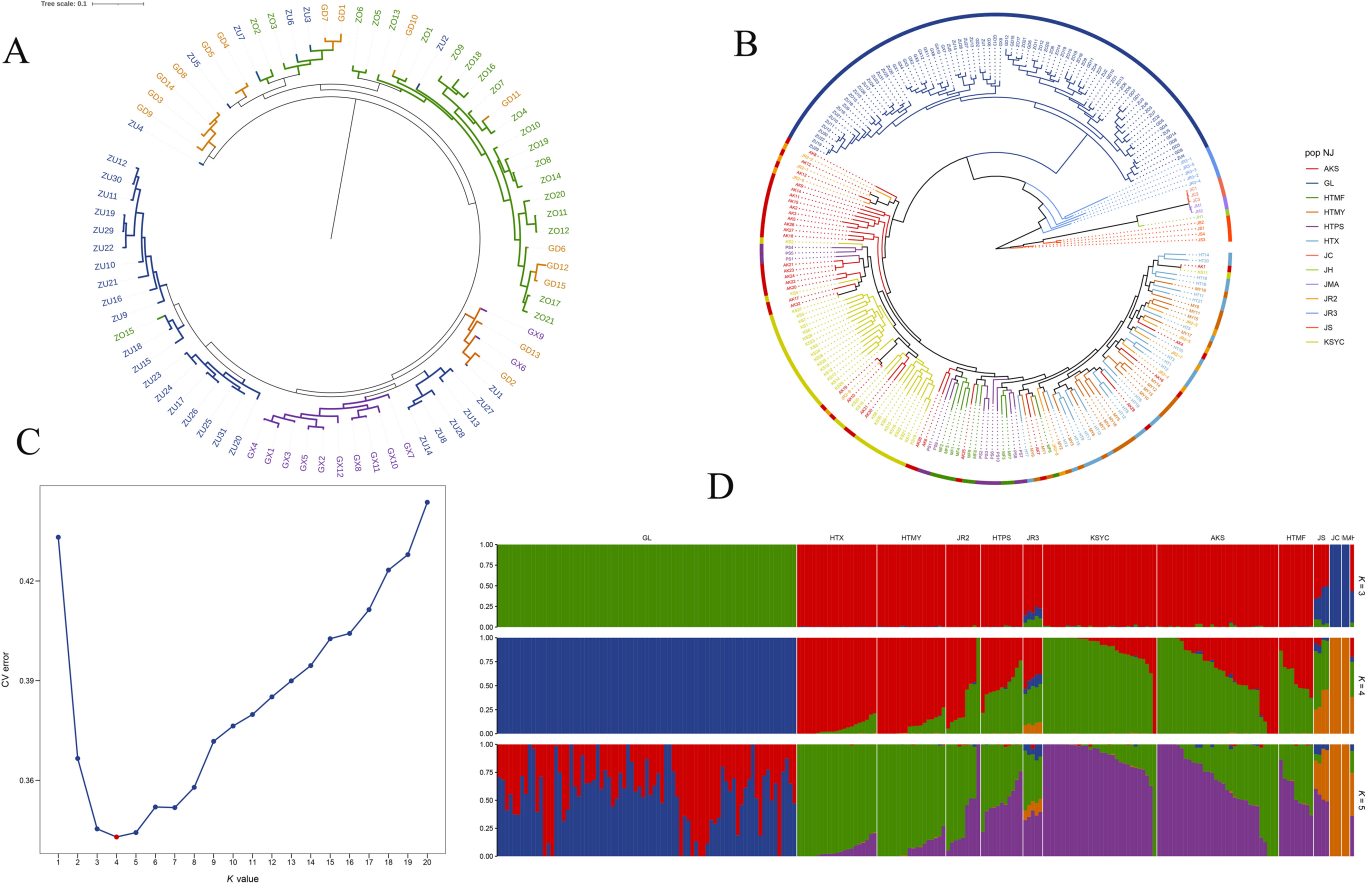
**(A)** Phylogenetic dendrogram of 79 wild walnut samples from Xinjiang. ZU represents the main valley, ZO represents the middle valley, GD represents the eastern valley, and GX represents the western valley. **(B)** Phylogenetic tree of 224 walnut samples constructed using the neighbor-joining method. Each branch represents a sample, with the junction of any two neighboring branches forming a node. The most basal node at the very base of tree is called the root node and represents the inferred ancestor of all extant lineages. Branches representing closely related species are labeled with the same color. **(C)** Cross-validation error for different K-values in a population structure analysis. The optimal K-value, with the smallest error, is marked in red. **(D)** Population structure diagrams (for K = 3, 4, and 5) created using an unsupervised maximum likelihood clustering algorithm in *ADMIXTURE*. Each color represents a distinct genetic cluster, with individuals represented by vertical bars that are subdivided to represent the proportion of an individual’s ancestry from each of the K genetic clusters. The optimal K-value was assessed by testing K values of 1 to 20, with K = 4 minimizing the cross-validation error. Population codes: GL (wild walnuts, incl. Glzu, Glzo, Gld, Glx); HTX, HTMY, HTPS, HTMF, KSYC (ancient seedlings); AKS (cultivated AK1-AK17, AK32; ancient seedlings AK18-AK31); JC, JH, JMA, JR2, JR3, JS (24 publicly available accessions).

To infer the phylogenetic relationships of wild walnuts in Xinjiang, we constructed a phylogenetic dendrogram based on genetic data from 79 samples (**Figure 4A**). In the dendrogram, six subpopulations could be distinguished within the wild walnut population, with samples from the east valley, the main valley, the middle valley, and the west valley forming four unique, independent subpopulations. The other two subpopulations were mostly comprised of samples from intersection zones between the valleys; one subpopulation included samples from the intersection of the east, main, and middle valleys, while the second included samples from the intersection of the east, main, and west valleys. Genetically, walnut trees located in the intersection zones were a mixture of the four valley subpopulations. Comparing subpopulations, the east valley subpopulation localized to the base of the dendrogram and was the first to diverge from the remaining subpopulations; the main, middle, and west valley subpopulations each formed three separate branches. The four core subpopulations likely evolved independently due to topographic barriers (i.e., steep slopes); however, post-glacial expansion led to gene flow within the intersection zones, forming two genetic “transition zones”. Individuals within valleys were tightly clustered on the phylogenetic tree, while different valleys were clearly segregated from each other, with valleys acting as genetic isolation barriers at the local scale.

To further explore how Xinjiang wild walnuts are related to other walnut accessions, the neighbor-joining method was used to construct a phylogenetic tree based on the SNP set (5,679,611 high-quality SNPs) created for all 224 walnut accessions (**Figure 4B**). In the dendrogram, walnut cultivars and landraces clustered into one large group that was distinct from that formed by the wild walnuts. Walnut landraces from Kashgar were most closely related to AK10, AK19, AK30, and AK31. Most of the walnut cultivars were most closely related to one another, although a few cultivars clustered with landraces from Hotan and Kashgar. Landraces from Hotan clustered into a single group which also contained some cultivars (AK1, AK4, AK6, AK7, and AK16). In the tree, the 24 accessions representing other walnut taxa were distantly related to the 200 accessions from Xinjiang, with the exception of the JR2 samples. For example, samples JR2-1, JR2-2, JR2-6, and JR2–9 were closely related to cultivated accessions from the Aksu region of Xinjiang, while JR2-3, JR2-4, JR2-5, JR2-7, and JR2–8 were closely related to walnut landraces from Hotan.

The maximum likelihood estimation of population structure identified four ancestral populations (i.e., K = 4) among the 224 walnut accessions (which comprised walnut cultivars [n = 18], landraces [n = 103], wild individuals [n = 79], and other germplasm [n = 24]) (**Figure 4D**). The genetic uniqueness of the Xinjiang wild walnuts was unequivocally supported by population structure analysis. At the optimal K = 4 (minimized cross-validation error; **Figure 4C**), they formed a distinct ancestral group, clearly differentiated from the admixed cultivars and landraces. This pattern of distinctness held true across multiple K-values (for K = 1 to K = 20; **Supplementary Figure S2**).

### 3.4 Principal component analysis and kinship analysis

**Figure 5:**
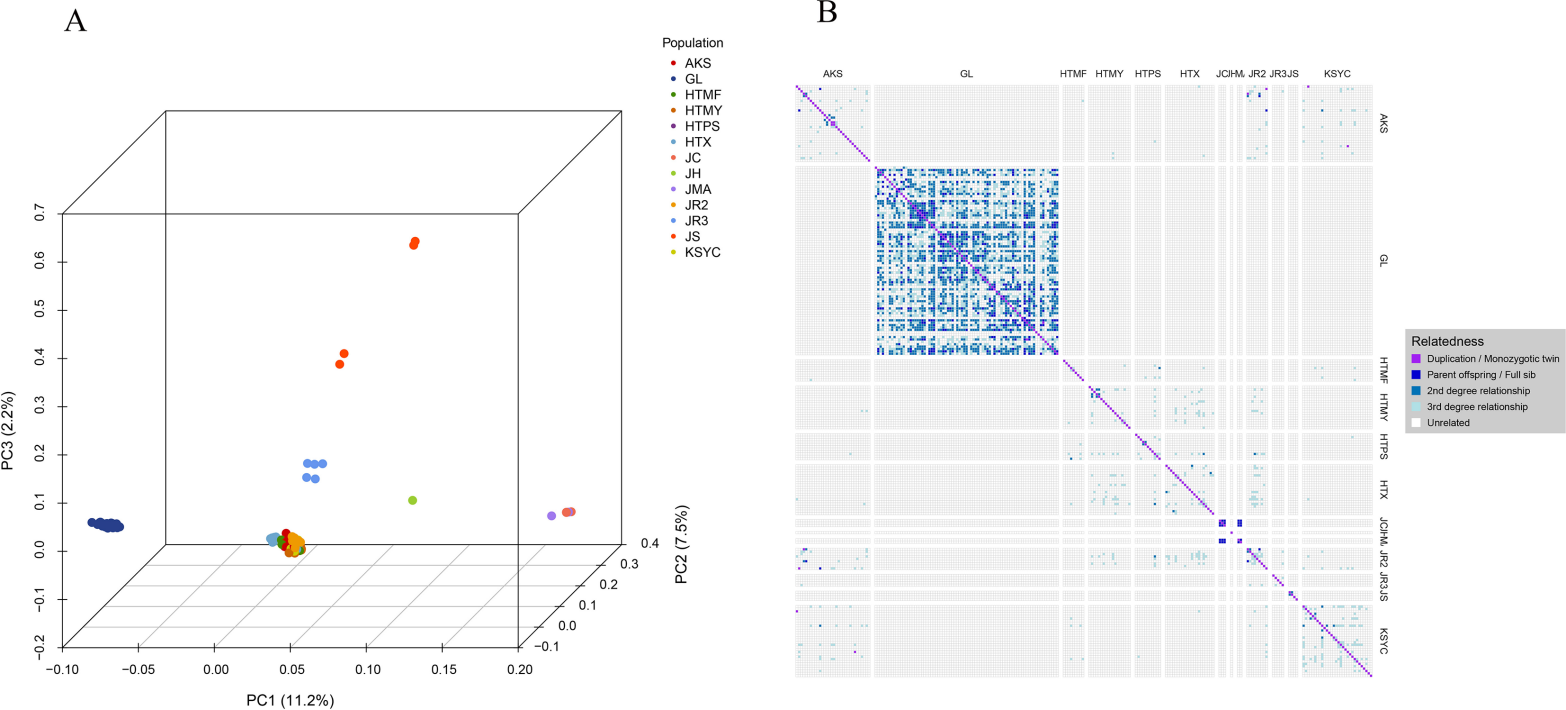
**(A)** Principal component analysis plot showing the first three principal components. Each dot in the plot represents a single sample, with dots colored to indicate population membership. Individuals that are closely related genetically are clustered together in the plot. **(B)** Kinship plot illustrating relationships between samples as estimated using pairwise SNP comparisons. Cells in the plot represent individual two-sample comparisons, with colors indicating the kinship rank of each comparison. Population codes: GL (wild walnuts, incl. Glzu, Glzo, Gld, Glx); HTX, HTMY, HTPS, HTMF, KSYC (ancient seedlings); AKS (cultivated AK1-AK17, AK32; ancient seedlings AK18-AK31); JC, JH, JMA, JR2, JR3, JS (24 publicly available accessions).

Principal component and kinship analyses confirmed the genetic distinctness of the Xinjiang wild walnuts. In the PCA, the first three principal components collectively explained 20.9% of the genetic variance. The wild walnuts formed a tight, distinct cluster that was clearly separate from the cultivars, landraces, and the 24 additional accessions (**Figure 5A**). This clear separation was further supported by kinship analysis, which revealed strong genetic cohesion within the wild group and distant relationships with all other populations (**Figure 5B**).

### 3.5 Selective sweep analysis

**Figure 6:**
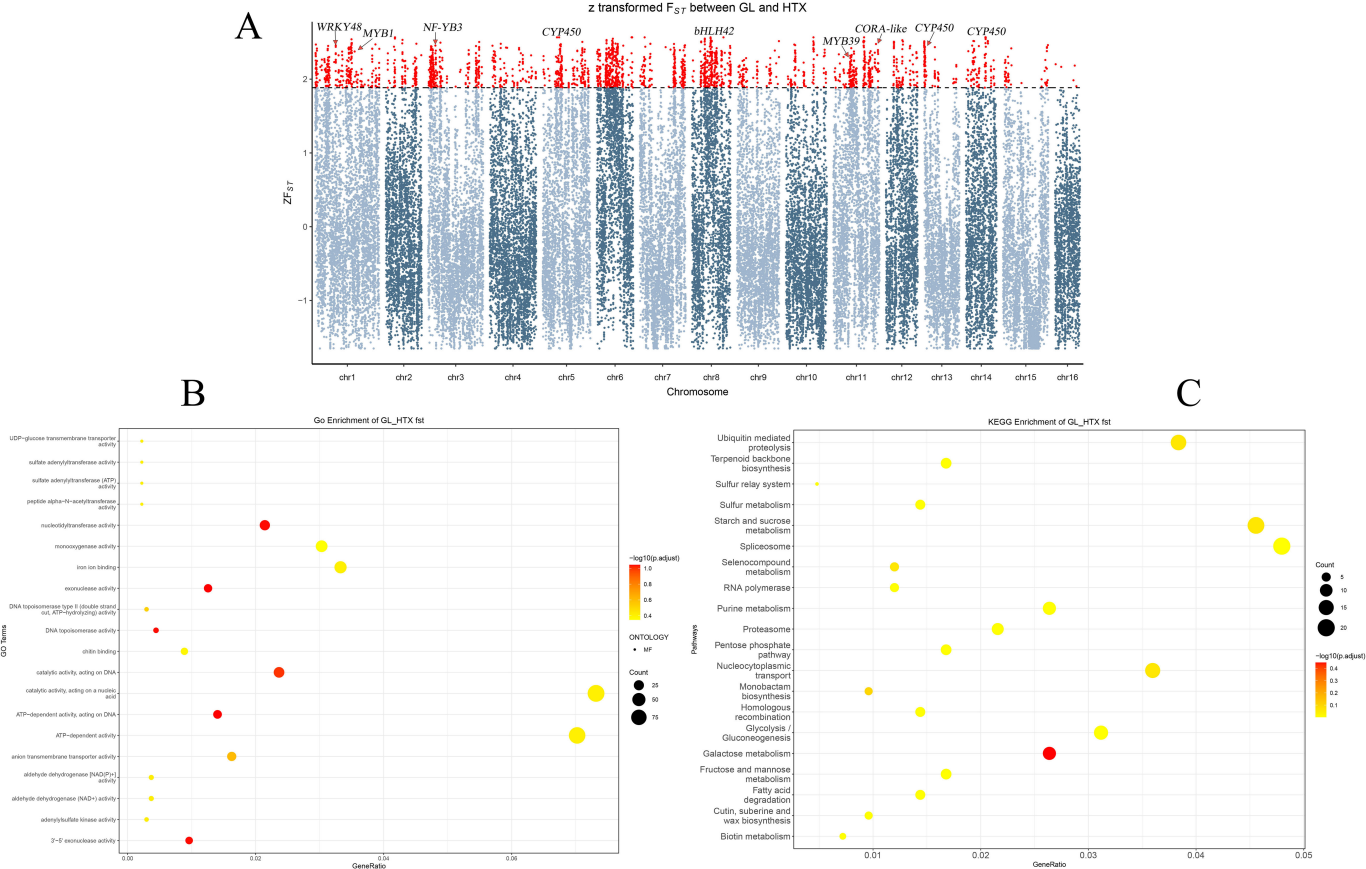
**(A)** Manhattan Plot of Z-Transformed *F*_ST_ Between Wild (GL) and Landraces (HTX) Walnut Populations. The x-axis represents the genomic coordinates across all 16 chromosomes. The y-axis shows the Z-transformed *F*_ST_ values, a measure of population genetic differentiation. The dashed horizontal line indicates the significance threshold (Default: top 5%). Red data points above this threshold represent genomic regions with significant selective sweep signals. Several candidate genes discussed in the study (e.g., GRP2, LEA16, NF-YA10, *CYP450*, COR) are highlighted near their respective genomic loci. The candidate genomic regions under positive selection may contribute to the stress resistance observed in the wild walnut (GL population). **(B)** GO enrichment of high *F*_ST_ Genes between GL and HTX Populations. The x-axis represents the gene ratio. The y-axis indicates the significantly enriched GO terms. Bubble color reflects the enrichment significance level, and bubble size corresponds to the number of candidate genes annotated in each GO term. **(C)** KEGG Enrichment of high *F*_ST_ Genes between GL and HTX Populations. The x-axis represents the gene ratio; the y-axis shows enriched KEGG pathways. Bubble color indicates enrichment significance, and bubble size corresponds to the number of genes in each pathway.

A selective sweep analysis based on *F*_ST_ values was performed between the Xinjiang wild and the ancient seedling populations of walnut to identify genes under strong selection (**Figure 6A**). Using the top 5% *F*_ST_ as a cutoff, we identified 28 candidate genes that may have contributed to local adaptation. Notably, several core transcription factors and enzyme-encoding genes intimately involved in disease resistance were under selection, indicating an enhanced immune system in the Xinjiang wild population. These included: WRKY transcription factors (Eulgem and Somssich, 2007; Chen et al., 2017) such asWRKY41 (JreChr03G11438), WRKY48 (JreChr01G10474),WRKY29 (JreChr11G11287), WRKY69 (JreChr08G10036), WRKY3 (JreChr03G10701), and WRKY23 (JreChr08G10021), which act as central regulators of plant immunity, extensively participating in salicylic acid and jasmonic acid signaling pathways; MYB transcription factors (Ma and Constabel, 2019; Liu et al., 2024) including MYB1 (JreChr01G13373), MYB4 (JreChr01G12459), MYB39 (JreChr11G10859), andMYB83 (JreChr13G11560), which primarily regulate the phenylpropanoid pathway for the synthesis of lignin and antimicrobial compounds; furthermore, the key jasmonate signaling transcription factor bHLH42 (JreChr08G10317) (Zhang et al., 2024) and several cytochrome P450 genes (Schuler and Werck-Reichhart, 2003; Nelson and Werck‐Reichhart, 2011) involved in the synthesis of defense compounds, such as CYP71D8 (JreChr14G10451), CYP72A219 (JreChr11G10191), CYP71AU50 (JreChr06G12173), and CYP71A6 (JreChr12G10259), were also selected.

**Table 1:** Genetic diversity metrics for 200 walnut accessions from the Xinjiang region in China.

Group	Number of samples	SNPs (N)	SNP/kb	π	Shannon diversity index
Wild walnuts	79	3,517,987	6.82	0.252	0.371
Landraces	103	3,707,223	7.19	0.308	0.447
Cultivars	18	3,813,489	7.39	0.305	0.457

Furthermore, the analysis also revealed the selection of genes associated with cold stress and abiotic stress response, consistent with adaptation to the native frigid climate. These primarily included: the cold and drought-regulated protein CORA-like (JreChr11G12006) (Jha et al., 2021); the nuclear transcription factor NF-YB3 (JreChr03G13037) (Lin et al., 2024), which enhances tolerance to drought and cold; cytochrome P450 genes (He et al., 2023) involved in hormone homeostasis to fine-tune the growth-defense trade-off, such as CYP94A1 (JreChr13G10978) and CYP714A1 (JreChr08G10054); as well as genes contributing to physical barrier formation, like CYP86A22 (JreChr11G11327) (Han et al., 2010) involved in cuticular wax synthesis and CYP704B1 (JreChr05G10202) (Kobayashi et al., 2021) involved in pollen wall development. These genes collectively constitute a multi-layered protective mechanism for the Xinjiang wild walnut to cope with low-temperature stress.

Based on the *F*_ST_ analysis between the GL wild and HTX ancient seedling populations, GO enrichment analysis (**Figure 6B**) revealed significant enrichment in molecular functions, including catalytic activity, acting on a nucleic acid (GO: GO:0140640), ATP-dependent activity (GO:0140657), catalytic activity, acting on DNA (GO:0140097), nucleotidyltransferase activity (GO:0016779), ATP-dependent activity, acting on DNA (GO:0008094), exonuclease activity (GO:0004527), 3’-5’ exonuclease activity (GO:0008408), DNA topoisomerase activity (GO:0003916).Among the cellular component category, significant enrichment was observed for the following terms: protein acetyltransferase complex (GO:0031248), acetyltransferase complex (GO:1902493), membrane-bounded organelle (GO:0043227), intracellular membrane-bounded organelle (GO:0043231), nucleus (GO:0005634), membrane-enclosed lumen (GO:0031974), organelle lumen (GO:0043233), intracellular organelle lumen (GO:0070013). These processes likely underlie the adaptive differentiation observed between the GL wild and HTX ancient seedling populations (comprehensive GO enrichment results are provided in **Supplementary Table 4**).

To further elucidate the biological functions enriched among genes with significant genetic differentiation (*F*_ST_) between the GL wild and HTX ancient seedling populations, KEGG pathway enrichment analysis (**Figure 6C**) revealed highly significant enrichment in pathways including galactose metabolism, starch and sucrose metabolism, the spliceosome, ubiquitin-mediated proteolysis, nucleocytoplasmic transport, glycolysis/gluconeogenesis, purine metabolism, monobactam biosynthesis, and selenocompound metabolism (detailed results of the KEGG enrichment analysis are provided in **Supplementary Table 5**).

## 4 Discussion

The publication of a high-quality walnut genome has facilitated research on related species of economic value (Zhang et al., 2020). Through genome annotation, genes associated with important agronomic traits have been identified in walnut, providing targets for molecular breeding programs aimed at developing varieties with enhanced disease resistance (Dai et al., 2025), cold tolerance (Han et al., 2024), and nut quality (Wang et al., 2022). Annotated genomic datasets have provided insights into gene family evolution and the molecular basis of pest/disease resistance in Juglandaceae species (Yan et al., 2021). As a rare wild plant resource in China, the Xinjiang wild walnut possesses significant ecological and genetic value. Its genome harbors a wealth of resistance gene resources (Xinjiang uygur autonomous region nature reserve research team, 1982), as exemplified by the identification of the cold-responsive *JfDREB1A* gene, which has been demonstrated to play a critical role in the species’ response to low-temperature stress (Han et al., 2023, 2024). However, compared to cultivated walnut samples, the unique genetic resources contained within Xinjiang wild walnut remain poorly characterized, and further in-depth studies of wild walnut samples are needed. In this study, whole genome resequencing was performed for 200 accessions of Xinjiang wild walnut, and a total of 5,679,611 high-quality SNPs were identified; this dataset represents a valuable resource for future studies of genetic differentiation in walnut.

The Xinjiang wild walnut population has a relatively restricted range within the Tianshan Mountains of China, concentrated in the Wild Walnut Valley Nature Reserve in Gongliu (Wang, 2011). Wild walnut trees are typically not seen in other regions of China, except for a dozen or so plants distributed within Huocheng County. Due to the distance between the Xinjiang population and wild walnuts in Kazakhstan, gene flow is unlikely to occur under natural conditions. It has been hypothesized that the low genetic diversity observed in the Xinjiang wild walnuts may be due to habitat fragmentation, resulting from environmental changes that led to isolation of the Tianshan Mountain populations. As a core element of biodiversity (Wei et al., 2021), genetic diversity directly determines a population’s adaptive potential (Taberlet et al., 2012; Du, 2023) and extinction risk threshold (Wang et al., 2023) Genetic diversity may be affected by anthropogenic disturbance (Lu et al., 2020; Perrino and Wagensommer, 2022), environmental changes (Provan, 2013), founder effects (Pellissier et al., 2016), gene flow (Taberlet et al., 2012), natural selection (Chhatre and Rajora, 2014), and population size (Crutsinger et al., 2008). As a result, the Tianshan populations may have experienced high levels of genetic drift or population bottlenecks, leading to a loss of heterozygosity, increased inter-individual similarity, and reduced overall genetic diversity (Allendorf et al., 2024). Phylogenetic analysis revealed that the Xinjiang wild walnut samples comprised six subpopulations, each exhibiting generally low genetic diversity. This finding is consistent with previous reports (Zhang et al., 2018; Li et al., 2024; Ye et al., 2024). Here, LD decayed relatively slowly within the wild walnut genomes, and samples were found to be closely related. This suggests the occurrence of historical bottlenecks or persistent small population effects, both of which may lead to heightened kinship among individuals. Frequent inbreeding within wild populations may have also led to declines in genetic diversity and increased homozygosity. Both wind-pollination and asexual reproduction, two features of walnut biology, may increase the likelihood of inbreeding, potentially decreasing the heterozygosity of alleles within a given population (Wang and Dang, 1999; Zhang et al., 2019c).

Population structure and kinship analyses demonstrated that the Xinjiang wild walnut population constitutes a unique and independent genetic group, showing no shared ancestry with other populations. Concordance between structural analysis (based on allele frequencies) and kinship analysis (based on genetic correlations) provides strong support for the finding that the Xinjiang wild walnut population constitutes a distinct genetic lineage. This conclusion of genetic distinctiveness was further supported by the results of the principal component analysis (PCA). It is worth noting that the first three principal components of PCA explained only 20.9% of the total variance cumulatively, which is comparable to the recent high-dimensional resequencing study of walnut (Wang et al., 2022). The low level of inter-valley differentiation results from the dispersion of genetic variance across a large number of genome-wide SNPs, each with a minimal effect, thereby distributing the variation among numerous low-effect loci. The close phylogenetic relationship between JR2 and some Xinjiang cultivated and landrace walnuts suggests they may share a common genetic background. In contrast, the significant differentiation between all 24 publicly available accessions and the Xinjiang wild walnuts further demonstrates that this wild population represents a unique evolutionary lineage, likely maintained in genetic isolation due to geographic barriers and limited gene flow.

Selective sweep analysis highlights genetic adaptations in Xinjiang wild walnuts supporting environmental resilience (Kajino et al., 2022). Strong selection signals in disease-resistant transcription factors such as WRKY (Caarls et al., 2015) and MYB families (Jiao et al., 2025) (e.g., *WRKY41* and *MYB4*) indicate enhanced immune signaling and phenylpropanoid metabolism, significantly improving resistance to pathogens. Meanwhile, genes such as *NF-YB3* (Lin et al., 2024) and *cytochrome P450 genes* (Huang et al., 2024) were under selection, demonstrating molecular adaptations to low-temperature stress. These results underscore the population’s unique genetic capacity for stress tolerance, highlighting its potential as a resource for resistance traits. The genetic differentiation observed in the Xinjiang wild walnut population was predominantly driven by biological functions conferring adaptation to natural environments, while in the cultivated walnut population, it was largely shaped by agronomic traits aligned with human selection, a pattern consistent with their independent evolutionary histories.

In a phylogenetic dendrogram of the Xinjiang samples, the 200 accessions were classified into three groups. The clear genetic separation of the wild walnuts stands in sharp contrast to the significant introgression observed between the ancient seedling walnuts from the Kashgar and Hotan regions. Walnut seeds are primarily dispersed by rodents (Zhang et al., 2017; Lenda et al., 2018), although anthropogenic dispersal also occurs. In walnut, pollination and seed dispersal mechanisms facilitate intra- and inter-population gene flow, thereby reducing genetic differentiation among populations (Nybom, 2004). The lower genetic differentiation indices of ancient solid walnut in the Hotan region as well as in the Kashgar region are inextricably linked to these factors. Habitat fragmentation (due to anthropogenic disturbance) may reduce gene flow and increase inbreeding in many tree species (Manners et al., 2013), but reductions in natural gene flow may be offset by human-mediated dispersal (e.g., of landraces). The remarkable genetic differentiation of wild walnut, consistent with its independent clustering in phylogenetic trees, suggests a unique evolutionary history characterized by long periods of isolation. The differing levels of genetic differentiation observed among these taxa suggest that the previously proposed evolutionary relationships between wild walnuts and various cultivated germplasms may require further validation with additional data.

The walnut ancestor was widely distributed across Eurasia from the Miocene onward (Erdei and Magyari, 2011; Yao et al., 2011; Zhang and Sun, 2011; Ivanov et al., 2012; Shatilova et al., 2014). However, the Last Glacial Maximum (LGM) caused a severe range contraction, forcing the species to persist in fragmented refugia, including Western Europe, Xinjiang, and a broad zone spanning from northeastern to southwestern China (Aradhya et al., 2017; Pollegioni et al., 2017; Feng et al., 2018; Li et al., 2024). Notably, genetic diversity within glacial refugia often declines due to repeated bottlenecks and post-glacial isolation (de Lafontaine et al., 2013; Gao and Gao, 2016). The discontinuous distribution of Xinjiang wild walnut is similar to that of the LGM glacial refuges. Genetic evidence also suggests that the Wild Walnut Valley may have been a naturally occurring glacial refuge (Feng et al., 2018; Li et al., 2024). Post-glacial colonies are known to exhibit lower genetic diversity than refugial populations (Hewitt, 1996, 1999). As population expansion occurs, frontier individuals will be derived from neighboring areas rather than from more distant ones (Hewitt, 1993); this may be one explanation for the low genetic diversity of wild walnuts in Xinjiang.

As humans resided in Xinjiang before the last glacial period (Finlayson, 2010; Stringer, 2012; García-Rodríguez et al., 2024), the genetic structure of contemporary wild walnut populations in Xinjiang cannot be explained solely by geography; anthropogenic factors must also be considered. For example, the flourishing of the Silk Road (French, 1998; Christian, 2000) facilitated extensive trade in walnuts between Asia and Europe (Aubaile, 2012), accelerating the spread of high-quality cultivated germplasm across the region. Archaeological evidence has dated widespread walnut remains along the Silk Road to the Han Dynasty (202 B.C.-A.D. 220) and earlier periods (Mishra, 2020), suggesting that the initial domestication of walnut predates the formation of transcontinental trade networks, such as the Silk Road. The center of domestication was likely located in western Central Asia or on the Iranian Plateau. Therefore, cultivated samples from Xinjiang (described here) may have been introduced via the Silk Road, with no direct relationship to local wild populations (not involved in domestication). Alternatively, wild walnuts in Xinjiang may represent de-domesticated ferals, originating from ancient cultivated varieties, with natural selection then enhancing cold- and drought-tolerance. Walnut species (and natural populations) have continued to evolve since the time of domestication, despite ongoing human activities (Miller and Gross, 2011).

Combining phylogenetic, population structure, and principal component analyses of 224 walnut samples, Xinjiang wild walnuts were found to be only distantly related to all Xinjiang cultivars and landraces, as well as other tested walnut germplasm. Wild Xinjiang populations have existed for a long time (surviving the last glacial maximum), and therefore may contain unique diversity potentially useful for crop improvement efforts, particularly for climate adaptation. The unique genetic identity of the Xinjiang wild walnut makes it a valuable wild genetic reservoir. Its genome may harbor resistance genes to cold or specific diseases that have been lost in cultivated varieties. This holds significant potential value for future genetic improvement of walnuts using strategies such as selection or hybridization. Thus, future conservation efforts should prioritize the protection of existing wild walnut forests, which serve as a germplasm resource for specific adaptive traits. Wild populations might be best protected by adopting *in situ* conservation measures and utilizing targeted relocation as needed (Ye et al., 2024).

## 5 Conclusions

In this study, the genetic structure of wild walnut populations and cultivated samples from Xinjiang, China, was analyzed using whole-genome resequencing. The study dataset revealed kinship relationships between Xinjiang wild walnuts and other walnut germplasm, and serves as an important resource for future walnut research and genomics-assisted breeding. In general, wild walnuts from Xinjiang had low genetic diversity and were significantly differentiated from other walnut samples, indicating a distant relationship to other walnut germplasm. However, the study included only a small number of samples of walnuts from outside Xinjiang. To more comprehensively characterize phylogenetic relationships among wild and cultivated walnuts, future studies should include more samples from areas surrounding Xinjiang. The construction of a cross-regional phylogenetic network would help elucidate the origins and evolutionary history of Xinjiang wild walnuts, helping to resolve the unique position of these samples within *Juglans*.

## Data Availability

Publicly available datasets were analyzed in this study. This data can be found here: SRR14888646, SRR5097507, SRR5097508, SRR9313661, SRR9313668, SRR9313665, SRR5192959, SRR5192958, SRR14430138, SRR14430165, SRR1443005, SRR14430040, SRR14430587, SRR14430584, SRR14430588, SRR14430059, SRR14430582, SRR14430540, SRR14430606, SRR14430345, SRR14430324, SRR14430515, SRR14430334, SRR14430326.
