# Supplementary material for: Genetic analysis of wild walnuts in Xinjiang based on whole-genome resequencing
Source: Front Plant Sci. 2025 Dec 19;16:1645319. doi: 10.3389/fpls.2025.1645319 (PMC12757410; doi:10.3389/fpls.2025.1645319)
Supplement: Supplementary file 1 [file DataSheet1.pdf]

Supplementary Material

1 Supplementary Tables and Figures

1.1 Supplementary Figures

Supplementary Figure 1.

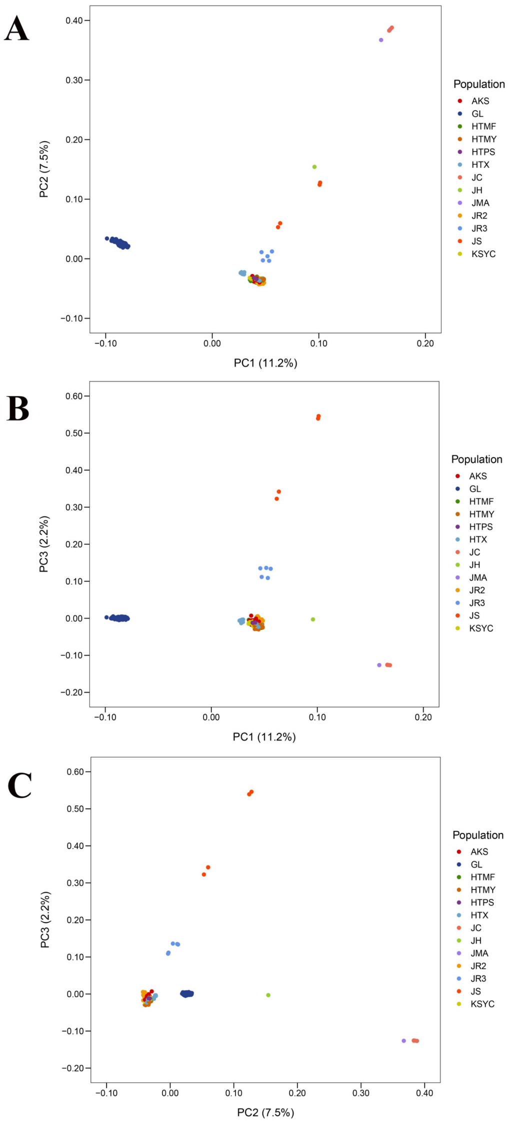

Figure 1. Principal component analysis plot showing the first three principal components. Each dot in the plot represents a single sample, with dots colored to indicate population membership. Individuals that are closely related genetically are clustered together in the plot. (**A**) PCA plot of

populations on PC1–PC2. (**B**) PCA plot of populations on PC1–PC3. (**C**) PCA plot of populations on PC2–PC3.

Supplementary Figure 2.

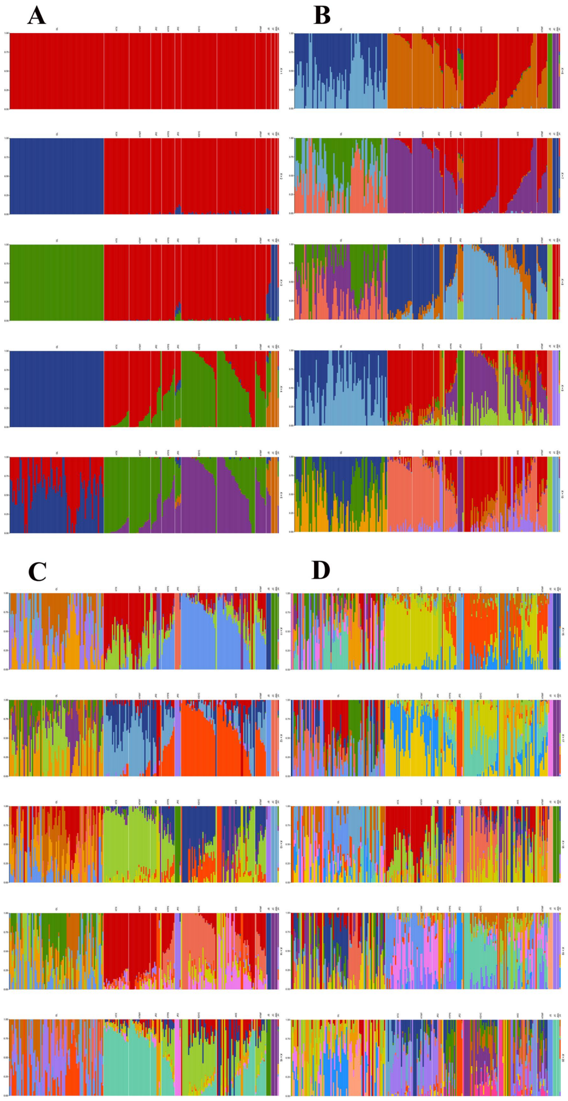

Figure 2. Population structure diagrams created using an unsupervised maximum likelihood clustering algorithm in *ADMIXTURE*. Each color represents an ancestral subpopulation (i.e., genetic cluster), with individuals represented by vertical bars that are subdivided to represent the proportion of an individual's ancestry from each of the K genetic clusters. The optimal K-value was assessed by testing K values of 1 to 20, with K = 4 minimizing the cross-validation error. (**A**) Population structure diagrams for K = 1-5. (**B**) Population structure diagrams for K = 6-10. (**C**) Population structure diagrams for K = 11-15. (**D**) Population structure diagrams for K = 16-20.
